# MicroRNAs and Presbycusis

**DOI:** 10.14336/AD.2017.0119

**Published:** 2018-02-01

**Authors:** Weiming Hu, Junwu Wu, Wenjing Jiang, Jianguo Tang

**Affiliations:** ^1^Department of Otolaryngology-Head and Neck Surgery, Zhejiang Provincial People’s Hospital, People’s Hospital of Hangzhou Medical College, Hangzhou 310014, China; ^2^Department of Otolaryngology-Head and Neck Surgery, Yiwu traditional Chinese Medicine Hospital, Yiwu 322000, China; ^3^Department of Otolaryngology-Head and Neck Surgery, Sir Run Run Shaw Hospital, Medical College of Zhejiang University, Hangzhou 310016, China.

**Keywords:** presbycusis, microRNAs, target gene, progressive hair cell degeneration, aging

## Abstract

Presbycusis (age-related hearing loss) is the most universal sensory degenerative disease in elderly people caused by the degeneration of cochlear cells. Non-coding microRNAs (miRNAs) play a fundamental role in gene regulation in almost every multicellular organism, and control the aging processes. It has been identified that various miRNAs are up- or down-regulated during mammalian aging processes in tissue-specific manners. Most miRNAs bind to specific sites on their target messenger-RNAs (mRNAs) and decrease their expression. Germline mutation may lead to dysregulation of potential miRNAs expression, causing progressive hair cell degeneration and age-related hearing loss. Therapeutic innovations could emerge from a better understanding of diverse function of miRNAs in presbycusis. This review summarizes the relationship between miRNAs and presbycusis, and presents novel miRNAs-targeted strategies against presbycusis.

Aging represents the accumulation of changes over time, encompassing physiological, psychological, and social changes. It is especially characterized by progressive degenerative changes in many living organs [1].

This process of aging is accompanied with increases in DNA damage, decreases in cellular water concentrations, reduction in mitochondrial function, ionic changes, vascular insufficiency, and decreased elasticity of cellular membranes [2, 3].

Aging is often accompanied by age-related degeneration of the auditory function (age-related hearing loss; ARHL) also known as presbycusis [4, 5]. Presbycusis is highly prevalent in developed countries and affects 25-40% of individuals older than 65 years of age. Because of the high prevalence of presbycusis , hearing difficulty becomes a common social and health problem and untreated hearing impairment contributes to depression, social isolation, and loss of self-esteem [5, 6]. Presbycusis is characterized by reduced hearing sensitivity and speech understanding in noisy environments, slowed central processing of acoustic information, and impaired localization of sound sources [5, 7].

Despite the hearing loss has been a serious problem in aging population, the pathogenesis of presbycusis remains poorly understood. According to previous studies, the cause of presbycusis is a combination of genetics, cumulative environmental exposures and pathophysiological changes related to aging. Most cases of presbycusis exhibit a mixture of these pathological changes. At present, antioxidants seem to be one of necessary but not enough preventive option for the presbycusis , in addition to hearing aid or surgical implant [8].

In the process of presbycusis , mitochondria play the key role in energy supply, biosynthesis and apoptosis of cells in the inner ear [9]. Oxidative stress, which is mainly caused by mitochondrial dysfunction, is known to play a causal role in presbycusis through the induction of apoptosis. MiRNAs are a group of endogenous, highly conserved and small single-stranded non-coding ribonucleic acids that can regulate gene expression through binding to partial base-pair complementarity with target messenger RNA (mRNA) ,leading to gene silencing [10]. miRNAs and other RNA interference have recently been reported to be present in mitochondria from several species [11].

Lin-4 was the first miRNA discovered in an observation of developmental mutations in Caenorhabditis elegans (*C. elegans*) in 1993 [10], but miRNAs did not show up in the inner ear until 2005, when the expression of miRNA was first discovered in the sensory organs and lateral line system in zebrafish [12]. The researchers found a cluster of miRNAs, including miRNA-96, miRNA-182 and miRNA-183, which were subsequently named as 183-miRNA family. Until now, the number of mature miRNA sequences has increased to 30,424 in 206 species [13]. The regulation of miRNA expression plays an important role in proliferation, differentiation and apoptosis [14]. A large number of miRNAs including the miR-29 family, miR-34 family, miR-200 family, miR-15/16, miR-17-92 cluster and miR-146a/b are considered to be involved in regulating cell senescence and death [15]. The first powerful evidence provided by Tsonis indicated that let-7 was intimately associated with hair cells regeneration in the vertebrate animals. It was the first time to associate hair cells regeneration with miRNAs in the vertebrate animal, implicating that a novel mechanism might be existed in hair cells regeneration and trans-differentiation. The results provided powerful evidence that miRNA expression was associated with hair cells regeneration [16]. Therefore, the down-regulated let-7 family might be related to dedifferentiation, which was a critical event for hair cell regeneration.

From then on, more and more miRNAs have been discovered in the inner ear, and approximately one third of all known miRNAs in the mouse were found to be expressed in its inner ear [17]. The seed sequence (nucleotides 2-8) of the miRNA is highly conserved in mammals and determine its target mRNA. But in most cases, the target mRNA has not been identified yet. The distinctive spatial and temporal expression pattern of each miRNA in the inner ear may help to reveal its targets and functions [18].

Recent studies have showed the variational expression of miRNAs in the inner ear of two mouse strains, C57BL/6J and CBA/J, which is the most widely used mouse model for the study of aging and age-associated diseases. During the process of age-related hearing loss, 111 and 71 miRNAs have been exhibited differential expression in the C57 and CBA mice, respectively [15]. Of all these miRNAs, the down-regulated miRNAs obviously exceeded the up-regulated ones. Besides the organ of Corti, Zhang also Identified the miRNAs involved in aging of the lateral wall of the cochlear duct. She found that a majority of down-regulated miRNAs are known to regulate pathways of cell proliferation and differentiation, while all up-regulated miRNAs are known regulators in the pro-apoptotic pathways [15, 19]. The down-regulated miRNAs, including the miR-183 family and miR-181 family, were involved in differentiation and proliferation [20]. Meanwhile the up-regulated miRNAs, including members of miR-29 family and miR-34 family, can affect genes with the potential to activate or enhance apoptosis pathways such as p53, p27 and Bcl2 [21].

## The miRNA-183 family

The miRNA-183 family contains miRNA-96, miRNA-182 and miRNA-183 and has become one of the most widely studied miRNAs for its roles about the inner ear. In both zebrafish and mouse, these three miRNAs are transcribed in the same orientation and are probably co-expressed in several neurosensory organs, including ear, nose, and eye, as they have been shown to have the similar expression forms in the zebrafish and the mouse [22]. The miRNA-183 family has been validated to be abundantly expressed in sensory neurons and hair cells in the inner ear in adulthood, and suggested that the cluster was important for cell specification and hair cell fate determination [17]. Subsequently, Xu and his colleagues have taken advantage of LNA (locked nucleic acid) probes to learn the miR-96/-182/-183 cluster, and demonstrated that the family was a specific cluster for sensory tissues, which is corresponding to previous study [23].

Expression patterns of miR-96, miR-182 and miR-183 in the developing inner ear have been studied recently [24]. These three miRNAs initially presented in the entire epithelium, then became limited to highly differentiated hair cells in both auditory organ and vestibula. The variation of spatial and temporal expression of these three miRNAs in mouse’s developing inner ear, with the obvious expression in differentiating hair cells, hinted that these miRNAs were of vital importance to hair cell differentiation and maturation. MiR-96 was the first miRNA found to be associated with deafness. The sequence of mature miR-96 is absolutely conserved within human, mouse, rat and fish [25]. Mencia first described two different point mutations in the seed region of MIR-96. The two different point mutations, located in the seed region of MIR-96, +13 G>A, and +14 C>A, respectively, were discovered in two unrelated Spanish families (locus DFNA50 on 7q32) and resulted in non-syndromic sensorineural, progressive hearing loss. So Mencia proposed that these mutations converted the regulatory function of miR-96 in keeping gene expression profiles in hair cells which was required for their normal function [26].

An ENU (N-ethyl-N-nitrosourea)-induced mouse with mutation of miR-96 was used to study the function of miRNA-96 in the inner ear [25]. The mutant, named diminuendo, has an A>T substitution in the seed region of miR-96. Sylamer analysis indicated that the mutation had both direct and indirect effects on expression of many genes. Utilizing the miRanda target predictor v3.0 and luciferase assay, five mRNAs were affirmed as miR-96 potential targets, and in non-inner-ear cell lines, their transcription was suppressed more significantly by wild-type miR-96 than by mutant miR-96 [26]. Then gene expression of all direct and indirect targets was observed, 36 were found to be down-regulated and 50 genes were found to be up-regulated. Of the down-regulated 36 genes, five indirect targets, which have neither wild-type nor mutant miR-96 target site, were attractive: Slc26a5 (prestin), Gfi1, Pitpnm1, Ocm (oncomodulin) and Ptprq. All these five genes were associated with deafness [27]. Prestin is a voltage-sensitive motor gene which plays an important role in the function of inner-ear hair cells and is linked with hair cell degeneration and hearing loss [28]. The mouse with Prestin removed has the change of hair cell morphology (such as short hair cells) and hair cell degeneration [29]. Gfi1 is expressed in hair cells, and the mouse lack of Gfi1 shows hair cell degeneration [30]. Oncomodulin is found to be expressed in outer hair cells and might play a role as a cytosolic calcium ion buffer [31]. Ptprq is necessary for maturity of the hair bundle, and is considered as a particular element of interstereocilial shaft connectors [27].

*In vitro*, Zhu Yan and his colleagues found that miRNAs could pass through gap junctions between native cochlear supporting cells to play a role in the cochlear development. They also found the deletion of Cx26 (Connexin26) but not Cx30 reduced miR-96 expression in the cochlea during postnatal development. The reduction is associated with the cochlear tunnel developmental disorder in Cx26 knockout (KO) mice. These data reveal that Cx26-mediated intercellular communication is required for cochlear development and that deficiency of Cx26 can impair miRNA-mediated intercellular genetic communication in the cochlea, which may lead to cochlear developmental disorders and eventually congenital deafness [32].

Another mutation in the seed region of miR-96-3p, +57 T>C, seated in the stem location of the miRNA precursor, is associated with hearing loss (HL) in an Italian family with autosomal dominant progressive HL. The +57(T>C) mutation replaced a nucleotide in the companion miR-96* is predicted to generate an enlarged RNA bulge in the secondary structure of pre-miR-96 hairpin, the Dicer cleavage site, and then interfere with Dicer processing. Finally, the novel mutation influences on the maturation of both miR-96 and miR-96* and leads to progressive HL [33]. Point mutations in seed sequences of miRNA-96 were shown in both mice and humans, with normal hearing at young ages, resulting in progressive hearing loss. The identified mutations suggest that the presence of wild-type miR-96 is necessary for survival of mature hair cells, but is not essential for the later development of hair cells in the inner ears. The seed sequences of miR-183 and miR-182 distinguish from the miR-96 seed only in the second nucleotide and in the eighth nucleotide, respectively. Thus, theoretically, miR-183 and miR-182 may partially neutralize for the deficiency of miR-96 and miR-96 may neutralize for the lack of miR-183 and miR-182 [18]. In most cases, miR-96 seed mutations may act as loss-of-function mutations. However, miR-96 seed mutations may also act as gain-of-function mutations. Such a gain of function has been mentioned in mice, because the point mutations may impact mRNAs which are not attacked by wild-type miR-183 family miRNAs [25].

## The MiRNA-34 family

MiR-34 is a markedly conserved miRNA, with identical seed sequence of orthologous in many organisms, including fly, C elegans, mouse, and human [34]. The miR-34 family in mammalians consists of three preserved miRNAs which are encoded by two different genes: miR-34a is generated by its own transcript, while miR-34b and miR-34c have a common elementary transcript. The exceeding resemblance among the three preserved miR-34 family members demonstrated that they may own the identical targets [19]. Using miRNA microarray analysis in both mouse and model cells, a lot of studies have shown that miR-34 expression increases with aging [35-37]. Over-expression of miR-34 in many cell processes results in cell cycle arrest. While enhancing expression of senescence-associated β-galactosidase (SA-β-Gal, the senescence marker), leads to cell senescence [38], and finally contributes to aging and age-related degenerative diseases. A recent study has showed that increased miR-34 has identified in the organ of Corti during presbycusis in the mouse model [15]. Several studies have demonstrated that decreases in the pharyngeal pumping rate or the rate of animal movement are obviously associated with declines in possibility of survival and can be applied to anticipate lifespan in *C. elegans* [39]. Worms carried the miR-34 loss-of-function mutant alleles gk437 and n4276, respectively, were used to prove the relationship between miR-34 and aging. Compared to wild-type N2 worms, the fast-pharyngeal pumping span of miR-34 mutants was significantly extended, and the fast moving span was obviously increased as well. MiR-34 mutants also showed an obvious increase in the maximum lifespan and the average lifespan of miR-34 mutants compared to wild-type animals. Based on these results, it was suggested that removing miR-34 prolongs the lifespan of *C. elegans*, which means delaying aging and age-related degenerative diseases [40]. To further prove whether autophagy, a lysosome-mediated digestive process, which is involved in organelle and protein degradation and plays a vital role in the control of aging and age-related degenerative diseases [41], is required for lifespan extension with mir-34 mutant alleles in worms. The influences of the yeast autophagy genes which had RNAi knockdown of *C. elegans* orthologs on miR-34 lifespan extension were tested. These genes included atg4.1 (yeast ATG4), bec-1 (yeast ATG6), and atg9 (yeast ATG9). RNAi with knockdown of atg4.1, bec-1,or atg9 significantly abrogated the lifespan extension phenotype in mir-34 mutants, but did not have impact on the lifespan of wild-type N2 worms, which is according with previous findings [42]. The results suggest that autophagy is essential for the mir-34 mutants induced lifespan extension and miR-34 may regulate the genes which are associated with the activity of autophagy and then control the *C. elegans* lifespan. To illuminate the specific roles of miR-34 in autophagy, whether miR-34 is participated in autophagic programs was investigated in vitro [40]. Hela cells and HEK293 cells, in which miR-34a expression was low or high, respectively, were used to elucidate the specific roles of miR-34 in autophagy processes. MiR-34a over-expression was established by transfection with miR-34a mimics in Hela cells, and miR-34a down-regulation was established by transfecting miR-34a inhibitors in HEK293 cells. By immunoblot analysis, Hela cells transfected with miR-34a mimics (100 nM) or HEK293 cells transfected with miR-34a inhibitors (100 nM) significantly affected SIRT1 protein expression, which has been verified as a direct target of miRNA-34a [43]. The result suggests that miR-34a function can be regulated by introducing miR-34a mimics in Hela cells or miR-34a inhibitors in HEK293 cells. By monitoring LC3 conversion, autophagic flux, a well-established marker for autophagy, is measured in the presence of lysosomal inhibitors [44]. The level of LC3-II was significantly decreased after over-expression of miR-34a in Hela cells and was significantly increased after the inhibition of miR-34a expression in HEK293 cells. Expression level of miR-34a could influence the level of Atg9A protein which was a protein implicated in the autophagosome formation has been confirmed to be essential at different steps of the autophagic machinery [45].

Recently, Pang Jiaqi and his team did a lot of work to present the circulating miR-34a level as a potentially useful biomarker for early detection of AHL. In their research, plasma miR-34a levels were significantly higher in patients with AHL compared with controls. However, miR-34a targets, such as SIRT1, Bcl-2, and E2F3 showed no correlation with AHL in humans [46]. Meanwhile Xiong Hao examined miR-34a/Sirtuin 1 (SIRT1)/p53 signaling in cochlear hair cells during aging, and the result of their work support a link between age-related cochlear hair cell apoptosis and miR-34a/SIRT1/p53 signaling, so they also believed this signaling may present an attractive target for the development of new drugs for AHL treatment [47].

Previous researches have shown that ectopic miR-34 resulted in a cell-cycle arrest and miR-34b/c repressed colony formation and proliferation in soft agar [48]. Several reports from different laboratories also have shown that the up-regulation of miR-34 family led to cell-cycle arrest and apoptosis, and that p53 is the direct target of the miR-34 family [49]. Target prediction algorithms predict there are 98 candidate targets for miR-34. In these mRNA, the down-regulated mRNAs showed a high level of miR-34 seed-matching sequences in their 3'-UTRs. Examples include CDK4/6, Cyclin E2, MET and Bcl-2, all of which are of importance for proliferation and differentiation [50]. It has been demonstrated that miR-34a lead to apoptosis through repressing silent information regulator 1 (SIRT1) expression in a p53-dependent pathway [43]. Further study verified that SIRT1 mRNA translation could be repressed by miR-34a through acting on a response section in the SIRT1 3'-UTR. WT HCT116 cells transfected with pre-miR-34a increased acetylated p53 and decreased SIRT1. In sharp contrast, HCT116 cells lacking p53 was not impacted by transfection with pre-miR-34a. These results suggest that miR-34a activates the p53 signaling pathway through p53 and the repression of SIRT1 by miR-34a is a part of a positive feedback loop which can further activate p53, once it has been activated [43].

Nevertheless, another study suggested that miR-34 played a neuro-protective function in the Drosophila brain through repressing expression of E74A, which is responsible for the neuro-degenerative disease [34]. In their study, Simon et al. found that 29 miRNAs expressed in the brain of adult Drosophila. Among all of 29 miRNAs, miRNA-34 was especially increased with aging while others decreased or kept a constant level with aging. Further research affirmed that miRNA-34 bind to the 3’UTR of the Eip74EF mRNA which is an important element of steroid hormone signaling pathways, whereas such pathways have been implicated in the lifespan regulation [51]. Aw et al. found that transcription of E74A, one of major protein isoforms of the Eip74EF gene, is in inverse proportion to the expression of miRNA-34. Up-regulation of the E74A expression lead to a dramatically shortened lifespan and premature neuro-degenerative disease in the adult [52]. This result verified that up-regulation of E74A significantly impacts normal aging, and one important role of miR-34 is to repress E74A and then to avert the deleterious activity of E74A in the adult. It was also observed that miR-34 mutants showed a defect of protein misfolding, a physiological process involved in many human neuro-degenerative diseases and aging [53]. All of these indicated that miRNA-34 had a neuro-protective role in the neuro-degenerative disease. Thus, the exact role of this miRNA in the neuro-degenerative disease, especially in presbycusis, has already been demonstrated but still need further exploration.

## The variant expression of microRNA induced by ROS lead to presbycusis

Seidman has showed that before cell dysfunction with cell senescence, activity of free oxygen radicals may lead to genetic and cellular alterations [2]. In animal models, the signs of oxidative stress distinctly increased in the cochlea of male CBA/J mice with aging and increased levels of p38 MAPK and JNK phosphorylation are observed in the cochleae of aging CBA/J mice [54]. These results suggest that reactive oxygen species (ROS) increases in the course of senescence of cochlea with aging, and multiple cell death pathways related to oxidative stress in aging mice are activated in the hair cells of the auditory organ [55]. High levels of reactive oxygen species (ROS) and oxidative stress are correlated to the age-related, the noise- and drug-induced hearing injury and loss [56].

Of importance, several studies indicate that excessive ROS build up is clearly the key factor in the pathogenesis of many stress-induced otological conditions, such as noise-induced hearing loss (NIHL), ototoxicity, tinnitus, as well as presbycusis [16]. To explore whether miRNAs are involved in ROS production in the ear, an in vitro cellular model system was used [57]. Tert-buty lhydroperoxide (t-BHP) was used to promote the generation of ROS in HEI-OC1 cells derived from the organ of Corti. Microarray analyses showed 35 miRNAs were found to be up-regulated, while 40 miRNAs were down-regulated in HEI-OC1 cells. In all these miRNAs, miR-200c, miR-29a and miR-17 were selected as specific examples and they were all up-regulated, while their predicted miRNA-target pairs, which were PTPN11, IGF-1 and PIK3R1, respectively, were down-regulated. A recent study has noted the different space and temporal expression of the IGF signaling elements (include IGF-1, PIK3R1, and PTPN11) during the development of inner ear and uncovered that IGF-1, via novel regulatory mechanisms, enhances sensory and neural cell survival and differentiation [58]. The study suggests that the variational expression of miRNA induced by ROS may cause the degeneration of Corti thus be involved in presbycusis [57].

## Other microRNAs involved in presbycusis

Previous study has showed that many miRNAs were differential expressed in the inner ear during presbycusis. MiRNA-124 was found that, through the conditional deletion of Dicer1 with Foxg1-cre which could result in miR-124 deletion, neurosensory cells of the ear appear a sharp degeneration after miR-124 is depleted within a few days, suggesting this miRNA is required for normal neuronal functions and maintaining normal audition [59]. Expression of miR-376 has been reported in the developing embryos and adult tissues, including the cochlea [60]. Another investigation suggests that the members of miR-376 cluster may play a role not only in regulating the development of the ganglia and sensory epithelia in the embryonic inner ear, but also in keeping normal function of spiral ganglion neurons and cells in the cochlear of adult mouse [61]. MiR-15a and miR-18a were found to be essential for the development of the inner ear and its sensory epithelia in zebrafish [60]. So far, hundreds of miRNAs have been identified in inner ears, and many miRNAs are expressed through adulthood, indicating that miRNAs implicate in both the development and maintenance of inner ear functions.

## MicroRNA and hair cell regeneration

The pathogenesis of presbycusis may be related with the degeneration of the cochlear cells including hair cells. Therefore, many researchers focus on regenerating hair cells for possible treatment of presbycusis. Unique miRNA expression has been linked to the stem cells differentiation. For example, in the invertebrate animal, miRNA in neoblasts of planaria has been associated with the capacity of regeneration [62]. Hair cell death was found in the inner ear of a urodele amphibian after treating with aminoglycoside antibiotic, but hair cells could be regenerated via trans-differentiation of the supporting cells [63]. Based on this work, Tsonis and his colleagues believed that miRNAs may play an important role during trans-differentiation, a process in which many tissue-specific genes are involved. To test this hypothesis, they carried out a study using the feature of regeneration of body parts and organs in adult newts and found that a large number of miRNAs were associated with hair cells and lens regeneration, especially the let-7 family [16]. It is the first time to contact hair cells regeneration with expression of miRNAs as novel regulators in the vertebrate animal, hinting that a novel mechanism might be implicated in hair cells trans-differentiation and regeneration. The results provided powerful evidence that miRNA expression was associated with hair cells regeneration and hinted that a possible physiological process might promote trans-differentiation and regeneration [16]. Therefore, the down-regulated expression of the let-7 family might implicate that they were regulators of dedifferentiation, which was a critical event for hair cell regeneration.

It has been demonstrated that the basilar papilla, the avian counterpart of the cochlea, has the ability to regenerate hair cells after hair cell loss [64]. Following injury, it is the supporting cells which produce new hair cells [65]. New hair cells can be first observed 4-5 days after the origin of exposure to an intense noise and undergo maturation so that they are actually indistinguishable from unaffected cells via 20-28 days after stimulus start [66]. Birds originally have increased hearing thresholds after ototoxic or acoustic injury, which ultimately restore nearly to normal audition, suggesting that regenerated hair cells have display function [67]. This recovery of function has been considered to result from not only new hair cells regeneration, but also restoration of those which have survived [68]. It has been shown that treatment with forskolin which is a potent adenylate cyclase activator that can enhance intracellular cAMP levels within the chick basilar papilla, causes an obvious and extensive proliferation of supporting cells, resulting in the regeneration of new hair cells [69]. It is believed that forskolin has a mitogenic effect in the mammalian vestibular system and treatment with forskolin could promote supporting cell to enter cell S-phase [70].

**Table1:** the miRNAs involved in regulating age-related hearing loss.

miRNA	Expression with aging	Possible target	Inference
let-7 family	downregulated	Tnfsf10/Caspase3/Birc5	Regulate dedifferentiation, promote hair cells regeneration
miRNA-183 family	downregulated	Slc26a5 (prestin)/ Gfi1/ Pitpnm1/ Ocm (oncomodulin) /Ptprq	Promote hair cell differentiation and maturation.
miRNA-34 family	upregulated	P53/E74A	Lead to cell-cycle arrest and apoptosis/protect neural cell in the neuro-degenerative disease
miR-200c, miR-29a and miR-17	upregulated	PTPN11/IGF-1/PIK3R1	Enhance sensory and neural cell survival and differentiation
miR-124	downregulated	Dicer1	Maintain normal neuronal functions and normal audition
miR-376	downregulated	phosphoribosyl pyrophosphate synthetase 1 (PRPS1)	Regulate the development of the ganglia and sensory epithelia in the embryonic inner ear; keep normal function of spiral ganglion neurons and cells in the cochlear
miR-15a, miR-18a	downregulated	Slc12a2/Cldn12/Bdnf	Regulate and control inner ear tissue differentiation and maintenance
miR-181a	downregulated	P27	Result in the production of new hair cells, and play an important role in auditory hair cell proliferation and regeneration
miR-29b	upregulated	SIRT1/PGC-1α	Induce cochlear hair cell apoptosis

It has been demonstrated that miR181a has a distinct pro-proliferative effect in myeloid leukemia cells [71]. This effect seems to be regulated in part by generating down-regulation of p27, which may be a barrier to promote hair cell regeneration in the vertebrate mammalian cochlea [72]. Frucht suggested over-expression of miRNA181a was essential to facilitate proliferation and regeneration in the normally quiescent avian auditory epithelium, endogenous miR181a conduce to the pro- proliferative function of forskolin in the avian inner ear and was a crucial regulator of forskolin in promoting proliferation in the avian inner ear. The conclusion hinted that overexpression of miR181a resulted in the production of new hair cells [73].

All of above consequence and data suggest that miRNA181a plays an important role in auditory hair cell proliferation and regeneration. Future studies will focus on the relationship between miRNA181a and its predicted targets and will probably illuminate the role of this miRNA in hair cell proliferation and regeneration.

Recently, Xue Tao and his colleagues found that there was a significant degeneration of cochlear hair cells and a higher expression of miR-29b in aged C57BL/6 mice compared with young mice. They also demonstrated the inhibition of miR-29b increased SIRT1 and PGC-1α expression, while it decreased apoptosis. Their founding supported a link between age-related cochlear hair cell apoptosis and miR-29b/SIRT1/PGC-1α signaling. As mentioned above, treatment of presbycusis is by hearing aid or surgical implant, but none of them was very effective. Interestingly, Tao’s founding now present an attractive pharmacological target for the development of novel drugs for the treatment of presbycusis [74].

We summarize the miRNAs that involved in regulating age-related hearing loss in our review.

## Conclusion

Presbycusis is one of the most common sensory disorders in the elderly population, and mitochondria act as the key role in the physiopathology of the inner ear cells. However, the molecular mechanism of cochlear hair cell apoptosis has not been fully understood yet.

Various research groups are seeking a sensitive and specific blood biomarker for diagnosis or early detection of presbycusis. Masoumeh Falah and his team demonstrate an upregulation of BAK1 gene expression and the BAK1/BCL2 ratio in peripheral blood samples from Iranian ARHI subjects, and they believed the gene expression changes in peripheral blood samples could be used as a rapid and simple biomarker for early detection of presbycusis [75]. Yang Dong and his team found that CCR3 and GILZ genes played an important role in the pathogenesis of presbycusis, probably by regulating chemokine receptors, T-cell apoptosis, or T-cell activation pathways. So, the CCR3 and GILZ genes can also be used as a potential biomarker for Presbycusis [76]. But unfortunately, their work didn’t involve in the miRNAs that related to these genes, and that might be the next challenge for our group. Meanwhile, circulating miR-34a level is the only miRNA may potentially serve as a useful biomarker for early detection of AHL until now [46].

To date, a great number of miRNAs have been verified having intimate relationship with presbycusis and emerging as promising therapeutic targets. These targets are as below: (1) the miRNA-183 family has become one of the most widely studied miRNAs for its role of hair cell fate determination. (2) MiRNA-181a is needed for stimulating proliferation in the avian basilar papillae and contributes to the regeneration of new hair cells [21]. But the precise function of the inner ear is still unknown. (3) Plasma miR-34a levels were significantly higher in patients with presbycusis compared with controls, indicating that it might be a useful biomarker for early detection of presbycusis [46]. Activation of miR-34a/Sirtuin 1 (SIRT1)/p53 signaling contributes to cochlear hair cell apoptosis, which may serve as a potential target for age-related hearing loss treatment [47]. Therefore, miR-34a in the neuro-degenerative disease, especially in presbycusis, has already been discovered but the mechanism still needs further exploration. (4) Let-7 family may be involved in regulating trans-differentiation of terminally differentiate cells in inner ear cells [16]. (5) A link was found between age-related cochlear hair cell apoptosis and miR-29b/SIRT1/PGC-1α signaling. This founding presents an attractive pharmacological target for the development of novel drugs for the treatment of presbycusis. (6) 95 and 60 miRNAs exhibited differential expressions in C57BL/6J and CBA/J mice during aging, respectively. miRNAs significantly up-regulated are pro-apoptotic, while those down-regulated are anti-apoptotic or pro-proliferative. It is possible that the underlying mechanism might be associated with a gradual and collective action of miRNAs regulation during aging [19].

Although the precise function of these miRNAs in regulating cellular senescence hasn’t been illuminated yet, and the development of new miRNAs-targeted strategy is currently hindered by the lack of reliable markers, it is still inspiring that clinical studies based on miRNAs against presbycusis would be explored. With the development of the RNA-sequence technology, more miRNAs related with the presbycusis could be identified. There is no doubt that the application of RNA-sequence technology will lead a paradigm shift in the understanding of the presbycusis, offer a new way to effectively predict the presbycusis and find functional miRNAs as attractive pharmacological targets. Thus, we believe that miRNAs will extend the avenue for the presbycusis treatment.
